# The influence of maternal prepregnancy weight and gestational weight gain on the umbilical cord blood metabolome: a case–control study

**DOI:** 10.1186/s12884-024-06507-x

**Published:** 2024-04-22

**Authors:** Xianxian Yuan, Yuru Ma, Jia Wang, Yan Zhao, Wei Zheng, Ruihua Yang, Lirui Zhang, Xin Yan, Guanghui Li

**Affiliations:** 1 Division of Endocrinology and Metabolism, Department of Obstetrics, Beijing Obstetrics and Gynecology Hospital, Capital Medical University, Beijing Maternal and Child Health Care Hospital, No. 251, Yaojiayuan Road, Chaoyang District, Beijing, 100026 China; 2 Department of Obstetrics and Gynecology, The Second Hospital of Jilin University, Changchun, 130041 Jilin China

**Keywords:** Maternal obesity, Gestational weight gain, Offspring health, Metabolites, Umbilical cord blood

## Abstract

**Background:**

Maternal overweight/obesity and excessive gestational weight gain (GWG) are frequently reported to be risk factors for obesity and other metabolic disorders in offspring. Cord blood metabolites provide information on fetal nutritional and metabolic health and could provide an early window of detection of potential health issues among newborns. The aim of the study was to explore the impact of maternal prepregnancy overweight/obesity and excessive GWG on cord blood metabolic profiles.

**Methods:**

A case control study including 33 pairs of mothers with prepregnancy overweight/obesity and their neonates, 30 pairs of mothers with excessive GWG and their neonates, and 32 control mother-neonate pairs. Untargeted metabolomic profiling of umbilical cord blood samples were performed using UHPLC‒MS/MS.

**Results:**

Forty-six metabolites exhibited a significant increase and 60 metabolites exhibited a significant reduction in umbilical cord blood from overweight and obese mothers compared with mothers with normal body weight. Steroid hormone biosynthesis and neuroactive ligand‒receptor interactions were the two top-ranking pathways enriched with these metabolites (*P* = 0.01 and 0.03, respectively). Compared with mothers with normal GWG, in mothers with excessive GWG, the levels of 63 metabolites were increased and those of 46 metabolites were decreased in umbilical cord blood. Biosynthesis of unsaturated fatty acids was the most altered pathway enriched with these metabolites (*P* < 0.01).

**Conclusions:**

Prepregnancy overweight and obesity affected the fetal steroid hormone biosynthesis pathway, while excessive GWG affected fetal fatty acid metabolism. This emphasizes the importance of preconception weight loss and maintaining an appropriate GWG, which are beneficial for the long-term metabolic health of offspring.

**Supplementary Information:**

The online version contains supplementary material available at 10.1186/s12884-024-06507-x.

## Background

The obesity epidemic is an important public health problem in developed and developing countries [1] and is associated with the emergence of chronic noncommunicable diseases, including type 2 diabetes mellitus (T2DM), hypertension, cardiovascular disease, nonalcoholic fatty liver disease (NAFLD), and cancer [2–4]. Maternal obesity is the most common metabolic disturbance in pregnancy, and the prevalence of obesity among women of childbearing age is 7.1% ~ 31.9% in some countries [5]. In China, the prevalence of overweight and obesity has also increased rapidly in the past four decades. Based on Chinese criteria, the latest national prevalence estimates for 2015–2019 were 34.3% for overweight and 16.4% for obesity in adults (≥ 18 years of age) [6].

Increasing evidence implicates overnutrition in utero as a major determinant of the health of offspring during childhood and adulthood, which is compatible with the developmental origins of health and disease (DOHaD) framework [7]. Maternal obesity and excessive gestational weight gain (GWG) are important risk factors for several adverse maternal outcomes, including gestational diabetes and hypertensive disorders, fetal death, and preterm birth [8–10]. More importantly, they have negative implications for offspring, both perinatally and later in life. Evidence from cohort studies focusing on offspring development confirms the relationship between maternal obesity/excessive GWG and offspring obesity programming [11–13]. Currently, there is no unified mechanism to explain the adverse outcomes associated with maternal obesity and excessive GWG, which may be the independent and interactive effects of the obese maternal phenotype itself and the diet associated with this phenotype. In addition to genetic and environmental factors, metabolic programming may also lead to the intergenerational transmission of obesity through epigenetic mechanisms.

Metabolomics, which reflects the metabolic phenotype of human subjects and animals, is the profiling of metabolites in biofluids, cells and tissues using high-throughput platforms, such as mass spectrometry. It has unique potential in identifying biomarkers for predicting occurrence, severity, and progression of diseases, as well as exploring underlying mechanistic abnormalities [14, 15]. Umbilical cord metabolites can provide information about fetal nutritional and metabolic health, and may provide an early window for detection of potential health issues in newborns [16]. Previous studies have reported differences in umbilical cord metabolite profiles associated with maternal obesity [17, 18]. However, the results were inconsistent due to differences in sample sizes, ethnicity and region, and mass spectrometry. In addition, most studies have not considered the difference in the effects of prepregnancy body mass index (BMI) and GWG on cord blood metabolites.

To investigate the relationship between early metabolic programming and the increased incidence of metabolic diseases in offspring, we studied the associations between elevated prepregnancy BMI/excessive GWG and umbilical cord metabolic profiles. Another purpose of this study was to explore whether there were differences in the effects of prepregnancy overweight/obesity and excessive GWG on cord blood metabolites.

## Methods

### Study population

This was a hospital-based, case control study that included singleton pregnant women who received prenatal care and delivered vaginally at Beijing Obstetrics and Gynecology Hospital, Capital Medical University, from January 2022 to March 2022. We selected 33 pregnant women with a prepregnancy BMI ≥ 24.0 kg/m^2^ regardless of their gestational weight gain as the overweight/obese group, 30 pregnant women with a prepregnancy BMI of 18.5–23.9 kg/m^2^ and a GWG > 14.0 kg as the excessive GWG group, and 32 pregnant women with a BMI of 18.5–23.9 kg/m^2^ and a GWG of 8.0–14.0 kg as the control group. The ages of the three groups were matched (± 1.0 years), and the prepregnancy BMIs of the excessive GWG and control groups were matched (± 1.0 kg/m^2^).

The inclusion criteria were women with singleton pregnancies, those aged between 20 and 45 years, those with full-term delivery (gestational age ≥ 37 weeks), those with a prepregnancy BMI ≥ 18.5 kg/m^2^, those without prepregnancy diabetes mellitus (DM) or hypertension, and those without gestational diabetes mellitus (GDM). The exclusion criteria were women with multiple pregnancies, those less than 20 years or more than 45 years old, those with a prepregnancy BMI < 18.5 kg/m^2^, those with prepregnancy DM, hypertension or GDM, and those without cord blood samples.

We classified pregnant women into BMI categories based on Chinese guidelines [19]: normal weight (prepregnancy BMI 18.5–23.9 kg/m^2^), overweight (prepregnancy BMI 24.0–27.9 kg/m^2^), and obese (prepregnancy BMI ≥ 28.0 kg/m^2^). GWG guideline concordance was defined by the 2021 Chinese Nutrition Society recommendations according to prepregnancy BMI. The upper limits of GWG for normal weight, overweight, and obesity were 14.0 kg, 11.0 kg, and 9.0 kg, respectively.

Ethical approval and written informed consent were obtained from all participants. The study has been performed according to the Declaration of Helsinki, and the procedures have been approved by the ethics committees of Beijing Obstetrics and Gynecology Hospital, Capital Medical University (2021-KY-037).

### Sample and data collection

Maternal and neonatal clinical data were collected from the electronic medical records system of Beijing Obstetrics and Gynecology Hospital. Maternal clinical characteristics included age, height, prepregnancy and predelivery weight, education level, smoking and drinking status during pregnancy, parity, conception method, comorbidities and complications of pregnancy, family history of DM and hypertension, gestational age, mode of delivery, and biochemical results during pregnancy. Prepregnancy BMI was calculated as prepregnancy weight in kilograms divided by the square of height in meters. GWG was determined by subtracting the prepregnancy weight in kilograms from the predelivery weight in kilograms. GDM was defined using the IAPDSG’s diagnostic criteria at 24 to 28^+6^ weeks gestation and the fasting glucose and 1- and 2-h glucose concentrations at the time of the oral glucose tolerance test (OGTT). Neonatal clinical characteristics included sex, birth weight and length. Macrosomia was defined as a birth weight of 4,000 g or more [20]. Low birth weight (LBW) was defined as a birth weight less than 2,500 g [21].

Umbilical cord blood samples were obtained by trained midwives after clamping the cord at delivery. Whole blood samples were collected in EDTA tubes, refrigerated for < 24 h, and centrifuged at 2,000 r.p.m. at 4 ℃ for 10 min. Plasma aliquots were stored at -80 ℃ until shipment on dry ice to Novogene, Inc. (Beijing, China) for untargeted metabolomic analysis.

### Untargeted metabolomic analyses

Ultrahigh-performance liquid chromatography tandem mass spectrometry (UHPLC‒MS/MS) analyses were performed using a Vanquish UHPLC system (Thermo Fisher, Germany) coupled with an Orbitrap Q Exactive™ HF mass spectrometer (Thermo Fisher, Germany) at Novogene Co., Ltd. (Beijing, China). Detailed descriptions of the sample preparation, mass spectrometry and automated metabolite identification procedures are described in the Supplementary materials.

### Statistical analysis

#### Clinical data statistical analysis

Quantitative data are shown as the mean ± standard deviation (SD) or median (interquartile range), and categorical data are presented as percentages. The Mann‒Whitney U test, chi-square test, and general linear repeated-measures model were used to assess the differences between the control and study groups when appropriate. A *P* value < 0.05 was considered statistically significant. All analyses were performed using Statistical Package of Social Sciences version 25.0 (SPSS 25.0) for Windows (SPSS Inc).

#### Umbilical cord metabolome statistical analysis

These metabolites were annotated using the Human Metabolome Database (HMDB) (https://hmdb.ca/metabolites), LIPIDMaps database (http://www.lipidmaps.org/), and Kyoto Encylopaedia of Genes and Genomes (KEGG) database (https://www.genome.jp/kegg/pathway.html). Principal component analysis (PCA) and partial least-squares discriminant analysis (PLS-DA) were performed at metaX. We applied univariate analysis (*T* test) to calculate the statistical significance (*P* value). Metabolites with a variable importance for the projection (VIP) > 1, a *P* value < 0.05 and a fold change (FC) ≥ 2 or FC ≤ 0.5 were considered to be differential metabolites. A false discovery rate (FDR) control was implemented to correct for multiple comparisons. The *q*-value in the FDR control was defined as the FDR analog of the *P*-value. In this study, the *q*-value was set at 0.2. For clustering heatmaps, the data were normalized using z scores of the intensity areas of differential metabolites and were plotted by the Pheatmap package in R language.

The correlations among differential metabolites were analyzed by cor () in R language (method = Pearson). Statistically significant correlations among differential metabolites were calculated by cor.mtest () in R language. A *P* value < 0.05 was considered statistically significant, and correlation plots were plotted by the corrplot package in R language. The functions of these metabolites and metabolic pathways were studied using the KEGG database. The metabolic pathway enrichment analysis of differential metabolites was performed when the ratio was satisfied by x/n > y/N, and the metabolic pathway was considered significantly enriched when *P* < 0.05.

## Results

### Demographic characteristics of study participants

The demographic and clinical characteristics of the three population groups enrolled in the study are summarized in Table 1. Mothers had no significant difference regarding their ages or gestational ages. Compared to the mothers in the excessive GWG and control groups, those in the prepregnancy overweight/obesity group had a significantly higher prepregnancy BMI (25.6 (24.5, 27.2) kg/m^2^). However, there was no significant difference in prepregnancy BMI between mothers in the excessive GWG group (20.3 ± 1.2 kg/m^2^) and mothers in the control group (20.6 ± 1.5 kg/m^2^). Mothers in the excessive GWG group had the highest GWG (17.0 (15.5, 19.1) kg) among the three groups. The mean GWG of the mothers in the prepregnancy overweight/obesity group was 12.9 ± 3.8 kg, which was similar to that of the control group (11.8 ± 1.5 kg). It was noteworthy that among the 33 prepregnancy overweight/obese pregnant women, 20 of them had appropriate GWG, 1 had insufficient GWG, and 12 had excessive GWG. The proportion of mothers who underwent invitro fertilization and embryo transfer (IVF-ET) in the prepregnancy overweight/obesity group (15.2%) was significantly higher than that in the excessive GWG and control groups. There were no statistically significant differences in the proportions of pregnancy outcomes among the three groups, including preeclampsia, premature rupture of membranes, postpartum hemorrhage, macrosomia, and LBW. The babies in the three groups showed no significant difference regarding their birth weights or lengths.

**Table 1:** Demographics characteristics of the study participants

	Overweight/obesity (*n* = 33)	Excessive GWG (*n* = 30)	Control (*n* = 32)
Maternal			
Age, years	32.5 ± 3.9	31.8 ± 4.5	31.4 ± 3.9
Education level, n (%)			
Secondary or lower	2 (6.1%)	2 (6.7%)	0
Tertiary	31 (93.9%)	28 (93.3%)	32 (100.0%)
Smoking during pregnancy, n (%)	0	0	0
Drinking during pregnancy, n (%)	0	0	0
Pre-pregnancy BMI, kg/m^2^	25.6 (24.5, 27.2)^a,b^	20.3 ± 1.2	20.6 ± 1.5
Parity, n (%)			
Primiparous	19 (57.6%)	19 (63.3%)	25 (78.1%)
Multiparous	14 (42.4%)	11 (36.7%)	7 (21.9%)
IVF-ET, n (%)	5 (15.2%) ^a, b^	0	0
Family history of HTN, n (%)	14 (42.4%) ^a^	1 (3.3%) ^c^	11 (34.4%)
Family history of DM, n (%)	4 (12.1%)	2 (6.7%)	5 (15.6%)
PCOS, n (%)	0	0	1 (3.1%)
Preeclampsia, n (%)	3 (9.1%)	0	0
Premature rupture of membrane, n (%)	14 (42.4%)	12 (40.0%)	8 (25.0%)
Postpartum hemorrhage, n (%)	6 (18.2%)	5 (16.7%)	2 (6.3%)
Gestation at delivery, week	39.6 ± 1.2	39.5 ± 0.9	39.7 ± 1.1
GWG, kg	12.9 ± 3.8^a^	17.0 (15.5, 19.1)^c^	11.8 ± 1.5
Mode of delivery, n (%)			
Spontaneous vaginal delivery	27 (81.8%)	25 (83.3%)	28 (87.5%)
Assisted vaginal delivery	6 (18.2%)	5 (16.7%)	4 (12.5%)
Neonatal			
Sex, n (%)			
Male	20 (60.6%)	13 (43.3%)	17 (53.1%)
Female	13 (39.4%)	17 (56.7%)	15 (46.9%)
Birth weight, g	3361.5 ± 381.8	3294.7 ± 342.5	3258.8 ± 371.0
Birth length, cm	50 (50, 50)	50 (50, 50)	50 (49, 50)
Macrosomia, n (%)	1 (3.0%)	0	1 (3.1%)
LBW, n (%)	0	0	0

*Abbreviations*: *GWG* gestational weight gain, *BMI* body mass index, *HTN* hypertension, *DM* diabetes mellitus, *IVF-ET* in vitro fertilization and embryo transfer, *PCOS* polycystic ovarian syndrome, *LBW* low birth weight

^a^Overweight/obesity vs. excessive GWG *P* < 0.05

^b^Overweight/obesity vs. control *P* < 0.05

^c^Excessive GWG vs. control *P* < 0.05

The biochemical parameters of the mothers during pregnancy are shown in Table 2. The levels of triglyceride (TG) and uric acid (UA) of mothers in the prepregnancy overweight/obesity group were significantly higher than those of the mothers in the excessive GWG and control groups in the first trimester. However, there was no significant difference in the blood glucose and lipid levels in the second and third trimesters of pregnancy among the three groups.

**Table 2:** The biochemical parameters of the mothers during pregnancy

	Overweight/obesity(*n* = 33)	Excessive GWG (*n* = 30)	Control (*n* = 32)
The first trimester of pregnancy (8–14 weeks)			
GLU, mmol/L	4.59 ± 0.30	4.65 ± 0.27	4.57 ± 0.25
TCHO, mmol/L	4.24 ± 0.55	4.17 (3.51, 4.40)	4.10 ± 0.76
TG, mmol/L	1.10 (0.92, 1.75)^a,b^	0.95 ± 0.30	0.93 (0.72, 1.21)
HDL-C, mmol/L	1.44 ± 0.29	1.53 ± 0.22	1.56 ± 0.25
LDL-C, mmol/L	2.36 ± 0.62	2.12 (1.96, 2.57)	2.14 ± 0.65
UA, μmol/L	253.6 ± 55.0^a,b^	212.5 ± 42.6	194.8 (176.2, 238.3)
HCY, μmol/L	6.30 (5.70, 6.70)	6.20 (5.35, 7.90)	6.11 ± 1.21
The second trimester of pregnancy (24–28 weeks)			
OGTT			
GLU (0h), mmol/L	4.47 ± 0.26	4.47 (4.32, 4.59)	4.34 ± 0.30
GLU (1h), mmol/L	7.52 ± 1.18	6.97 ± 1.61	7.54 ± 1.36
GLU (2h), mmol/L	6.25 ± 0.89	6.15 ± 1.01	6.33 ± 0.99
TCHO, mmol/L	5.60 ± 0.82	6.30 ± 1.45	6.02 ± 0.99
TG, mmol/L	2.29 ± 0.91	2.06 ± 0.60	1.87 (1.59, 2.34)
HDL-C, mmol/L	1.82 ± 0.28^a^	2.07 ± 0.39	2.04 ± 0.34
LDL-C, mmol/L	3.05 ± 0.83	3.39 (2.84, 3.94)	3.29 ± 0.95
The third trimester of pregnancy (32–34 weeks)			
GLU, mmol/L	4.25 (4.12, 4.62)	4.42 ± 0.40	4.31 ± 0.32
TCHO, mmol/L	6.15 ± 1.06	6.83 ± 1.37	6.77 (5.78, 7.69)
TG, mmol/L	2.91 ± 0.96	2.43 (2.13, 3.12)	2.70 (2.40, 3.17)
HDL-C, mmol/L	1.88 ± 0.30	1.97 ± 0.33	2.01 ± 0.38
LDL-C, mmol/L	3.37 ± 1.04	3.58 (3.21, 4.46)	3.80 ± 1.02
UA, μmol/L	264.0 ± 46.1	249.1 (228.7, 310.4)	244.4 ± 44.7
HCY, μmol/L	5.10 (4.60, 5.60)	5.45 (5.10, 6.33)	5.91 ± 1.24

*Abbreviations*: *GWG* gestational weight gain, *GLU* glucose, *TCHO* total cholesterol, *TG* triglyceride, *HDL-C* high density lipoprotein-cholesterol, *LDL-C* low density lipoprotein-cholesterol, *UA* uric acid, *HCY* homocysteine, *OGTT* oral glucose tolerance test

^a^Overweight/obesity vs. excessive GWG *P* < 0.05

^b^Overweight/obesity vs. control *P* < 0.05

### PCA and PLS-DA analysis of cord blood metabolites

**Fig. 1:**
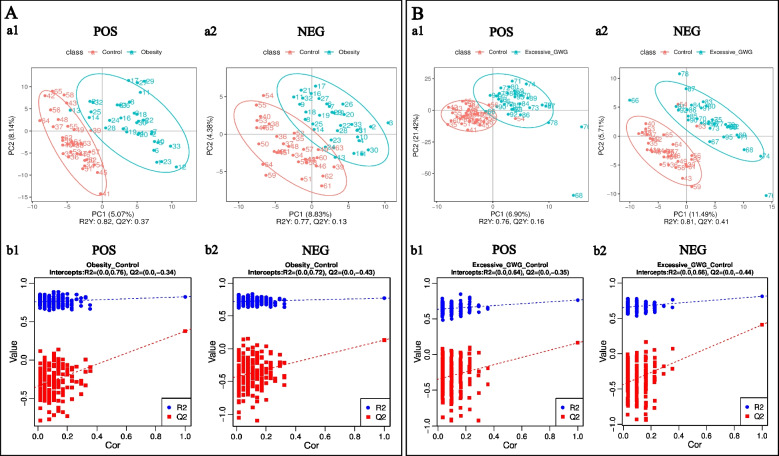
PLS-DA of identified cord blood metabolites. **A** the prepregnancy overweight/obesity group vs. the control group; **B** the excessive GWG group vs. the control group. (a) PLS-DA score. The horizontal coordinates are the score of the sample on the first principal component; the longitudinal coordinates are the score of the sample on the second principal component; R2Y represents the interpretation rate of the model, and Q2Y is used to evaluate the predictive ability of the PLS-DA model, and when R2Y is greater than Q2Y, it means that the model is well established. (b) PLS-DA valid. Horizontal coordinates represent the correlation between randomly grouped Y and the original group Y, and vertical coordinates represent the scores of R2 and Q2. (1) POS, positive metabolites; (2) NEG, negative metabolites

Functional and taxonomic annotations of the identified metabolites included the HMDB classification annotations, LIPID MAPS classification annotations, and KEGG pathway annotations. Those cord blood metabolites included lipids and lipid-like molecules, organic acids and their derivatives, and organoheterocyclic compounds, which were mainly involved in metabolism. To better understand the structure of the cord blood metabolome in cases versus controls, we used unsupervised PCA to identify metabolites contributing the most to observed differences in the dataset. PCA did not clearly separate the three groups. We next used PLS-DA to identify metabolites that were predictive of case versus control status. PLS-DA clearly distinguished the cases from the controls (Fig. 1), the prepregnancy overweight/obesity group vs. the control group (R2Y = 0.82, Q2Y = 0.37; R2Y = 0.77, Q2Y = 0.13, respectively) (Fig. 1A), and the excessive GWG group vs. the control group (R2Y = 0.76, Q2Y = 0.16; R2Y = 0.81, Q2Y = 0.41) (Fig. 1B).

### Maternal prepregnancy overweight/obesity

**Fig. 2:**
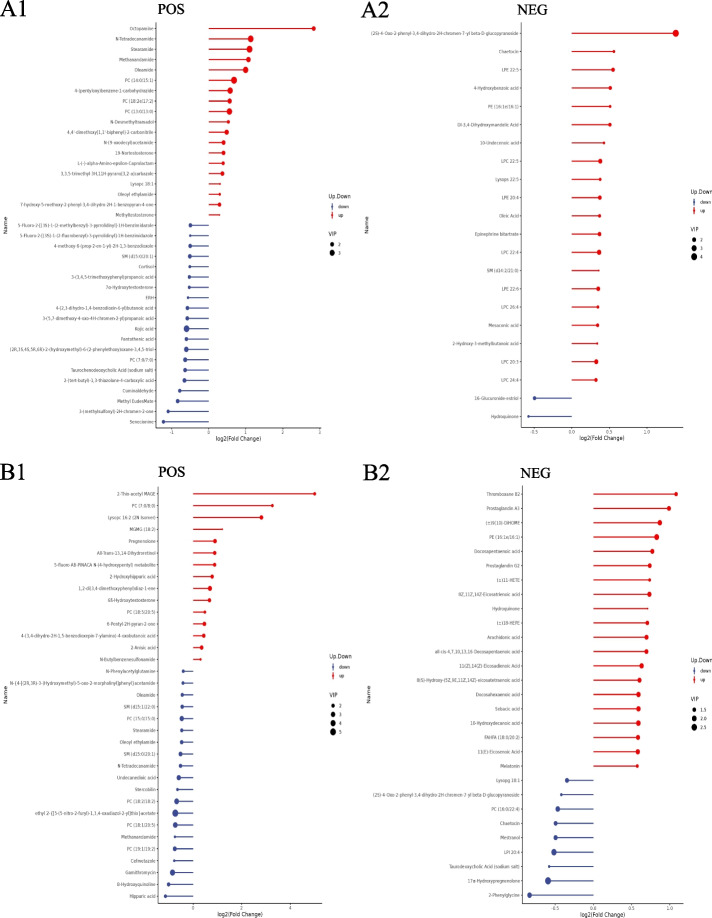
Stem plots of differential cord blood metabolites. **A** the prepregnancy overweight/obesity group vs. the control group; **B** the excessive GWG group vs. the control group. (1) positive metabolites; (2) negative metabolites. Notes: The color of the dot in the stem plots represents the upward and lower adjustment, the blue represents downward, and the red represents upward. The length of the rod represents the size of log2 (FC), and the size of the dot represents the size of the VIP value

Screening differential metabolites according to a PLS-DA VIP > 1.0, a FC > 1.2 or < 0.833 and a *P* value < 0.05, a total of 106 cord blood metabolites (77 positive metabolites and 29 negative metabolites) differed between the prepregnancy overweight/obesity group and the control group. Compared with those in the control group, the levels of 46 metabolites (19 positive metabolites and 27 negative metabolites) were increased in the prepregnancy overweight/obesity group, among which octopamine was the metabolite with the largest increase, followed by (2S)-4-Oxo-2-phenyl-3,4-dihydro-2H-chromen-7-yl beta-D-glucopyranoside, N-tetradecanamide, stearamide, and methanandamide (Fig. 2A). Compared with the control group, in the prepregnancy overweight/obesity group, there were 60 metabolites (58 positive metabolites and 2 negative metabolites) with reduced concentrations, among which senecionine was the metabolite with the largest decrease, followed by 3-(methylsulfonyl)-2H-chromen-2-one, methyl EudesMate, cuminaldehyde, and 2-(tert-butyl)-1,3-thiazolane-4-carboxylic acid (Fig. 2A).

**Fig. 3:**
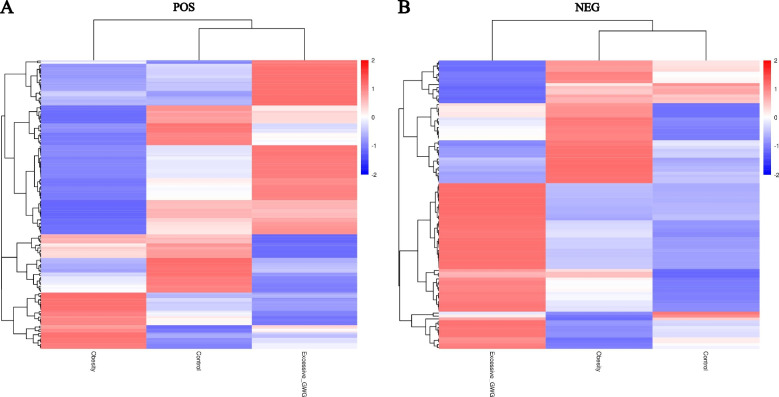
Clustering heat maps of differential cord blood metabolites of the three groups. **A** positive metabolites; **B** negative metabolites. Notes: Longitudinal clustering of samples and trans-verse clustering of metabolites. The shorter the clustering branches, the higher the similarity. Through horizontal comparison, we can see the relationship between groups of metabolite content clustering

**Fig. 4:**
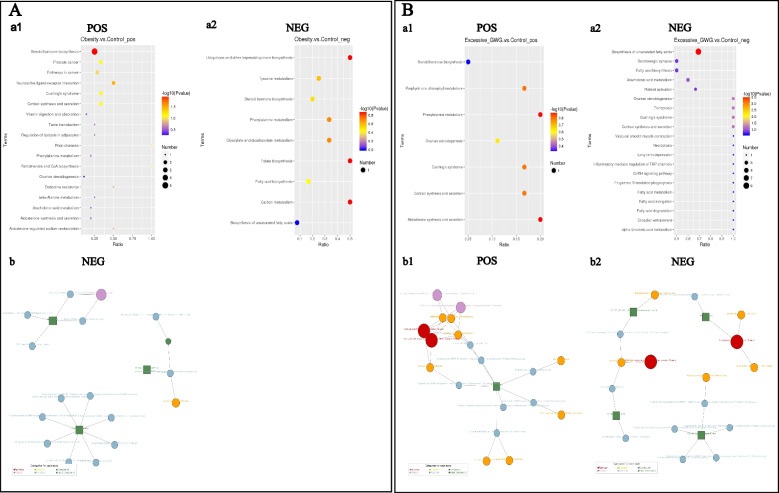
KEGG enrichment scatterplots (a) and net (b) of differential cord blood metabolites. **A** the prepregnancy overweight/obesity group vs. the control group; **B** the excessive GWG group vs. the control group. (1) positive metabolites; (2) negative metabolites. Notes: (a) The horizontal co-ordinates in the figure are x/y (the number of differential metabolites in the corresponding metabolic pathway/the total number of total metabolites identified in this pathway). The value represents the enrichment degree of differential metabolites in the pathway. The color of the point rep-resents the *P*-value of the hypergeometric test, and the size of the point represents the number of differential metabolites in the corresponding pathway. (b) The red dot represents a metabolic pathway, the yellow dot represents a substance-related regulatory enzyme information, the green dot represents the background substance of a metabolic pathway, the purple dot represents the molecular module information of a class of substances, the blue dot represents a substance chemical reaction, and the green square represents the differential substance obtained by this comparison

A hierarchical analysis of the two groups of differential metabolites obtained was carried out, and the difference in metabolic expression patterns between the two groups and within the same comparison was obtained, which is shown in Fig. 3. KEGG pathway analysis of differential cord blood metabolites associated with the prepregnancy overweight/obesity group versus the control group is shown in Table 3 and Fig. 4A. The metabolite enrichment analysis revealed that steroid hormone biosynthesis (*P* value = 0.01) and neuroactive ligand‒receptor interactions (*P* value = 0.03) were the two pathways that were most altered between the prepregnancy overweight/obesity group and the control group. 19 metabolites were distributed in the pathway of steroid hormone biosynthesis, and 4 metabolites were distributed in the pathway of neuroactive ligand‒receptor interactions. In the steroid hormone biosynthesis pathway, the levels of corticosterone, 11-deoxycortisol, cortisol, testosterone, and 7α-hydroxytestosterone were decreased in the prepregnancy overweight/obesity group relative to those in the control group. In the neuroactive ligand‒receptor interaction pathway, the level of cortisol was decreased and the levels of trace amines were increased in the prepregnancy overweight/obesity group relative to the control group.

**Table 3:** KEGG pathway analysis of cord blood metabolites associated with the pre-pregnancy overweight/obeisty group versus the control group

KEGG Pathway	Screened Metabolites	*P* value	Trend	Mode
Steroid hormone biosynthesis	Testosterone; 7α-Hydroxytestosterone; Cortisol; Corticosterone; Cortodoxone	0.01	Up	Positive
Neuroactive ligand-receptor interaction	Cortisol; Octopamine	0.03	Up	Positive
Cortisol synthesis and secretion	Cortisol; Cortodoxone	0.08	Up	Positive
Cushing’s syndrome	Cortisol; Cortodoxone	0.08	Up	Positive
Prostate cancer	Testosterone; Cortisol	0.08	Up	Positive
Pantothenate and CoA biosynthesis	Pantothenic acid	0.08	Up	Positive
Prion diseases	Corticosterone	0.08	Up	Positive

*Abbreviations*: *KEGG* Kyoto Encyclopedia of Genes and Genomes

### Maternal excessive GWG

A total of 109 cord blood metabolites (52 positive metabolites and 57 negative metabolites) differed between the excessive GWG group and the control group. Compared with the control group, in the excessive GWG group, there were 63 metabolites (15 positive metabolites and 48 negative metabolites) with increased concentrations, among which 2-thio-acetyl MAGE was the metabolite with the largest increase, followed by PC (7:0/8:0), lysopc 16:2 (2 N isomer), MGMG (18:2), and thromboxane B2 (Fig. 2B). Compared with the levels in the control group, the levels of 46 metabolites (37 positive metabolites and 9 negative metabolites) in the excessive GWG group were reduced, among which hippuric acid had the largest decrease, followed by 8-hydroxyquinoline, gamithromycin, 2-phenylglycine, and cefmetazole (Fig. 2B).

A hierarchical analysis of differential metabolites obtained in the two groups was carried out, and the difference in metabolic expression patterns between the two groups and within the same comparison was obtained, which is shown in Fig. 3. KEGG pathway analysis of the cord blood metabolites associated with the excessive GWG group versus the control group is shown in Table 4 and Fig. 4B. The metabolite enrichment analysis revealed that biosynthesis of unsaturated fatty acids was the most altered pathway between the excessive GWG and control groups (*P* value < 0.01). There were 13 metabolites distributed in the enriched pathway. The levels of docosapentaenoic acid (DPA), docosahexaenoic acid (DHA), arachidonic acid, adrenic acid, palmitic acid, stearic acid, behenic acid, lignoceric acid, and erucic acid were increased in the excessive GWG group relative to those in the control group.

**Table 4:** KEGG pathway analysis of cord blood metabolites associated with the excessive GWG group versus the control group

KEGG Pathway	Screened Metabolites	*P* value	Trend	Mode
Biosynthesis of unsaturated fatty acids	Stearic acid; Erucic acid; Palmitic acid; Lignoceric Acid; Docosanoic Acid; Docosahexaenoic acid; Arachidonic acid; Adrenic acid; Docosapentaenoic acid	0.00	Up	Negative
Ferroptosis	Arachidonic acid; Adrenic acid	0.06	Up	Negative
Ovarian steroidogenesis	17α-Hydroxypregnenolone; Arachidonic acid	0.06	Up	Negative
Cortisol synthesis and secretion	17α-Hydroxypregnenolone; Hydrocortisone	0.06	Up	Negative
Cushing’s syndrome	17α-Hydroxypregnenolone; Hydrocortisone	0.06	Up	Negative

*Abbreviations*: *KEGG* Kyoto Encyclopedia of Genes and Genomes, *GWG* gestational weight gain

## Discussion

Our present study found that both maternal prepregnancy overweight/obesity and excessive GWG could affect umbilical cord blood metabolites, and they had different effects on these metabolites. Regardless of their gestational weight gain, the umbilical cord blood of prepregnancy overweight and obese mothers had 46 metabolites increased and 60 metabolites decreased compared with the umbilical cord blood of mothers with normal body weight and appropriate GWG. Steroid hormone biosynthesis and neuroactive ligand‒receptor interactions were the two top-ranking pathways enriched with these metabolites. Compared with mothers with normal prepregnancy BMI and appropriate GWG, in mothers with normal prepregnancy BMI but excessive GWG, the levels of 63 metabolites were increased and those of 46 metabolites were decreased in umbilical cord blood. Biosynthesis of unsaturated fatty acids was the most altered pathway enriched with these metabolites.

There were many differential metabolites in the cord blood between the prepregnancy overweight/obesity group and the control group and between the excessive GWG group and the control group. However, the roles of most of these differential metabolites are unknown. The levels of stearamide and methanandamide were increased in the prepregnancy overweight/obesity group. Stearamide, also known as octadecanamide or kemamide S, belongs to the class of organic compounds known as carboximidic acids. Stearamide, which is increased in the serum of patients with hepatic cirrhosis and sepsis, may be associated with the systemic inflammatory state [22, 23]. Methanandamide is a stable analog of anandamide that participates in energy balance mainly by activating cannabinoid receptors. Methanandamide dose-dependently inhibits and excites tension-sensitive gastric vagal afferents (GVAs), which play a role in appetite regulation [24]. In mice fed a high-fat diet, only an inhibitory effect of methanandamide was observed, and GVA responses to tension were dampened [24, 25]. These changes may contribute to the development and/or maintenance of obesity. Moreover, methanandamide can produce dose-related hypothermia and attenuate cocaine-induced hyperthermia by a cannabinoid 1-dopamine D2 receptor mechanism [26].

Metabolomic pathway analysis of the cord blood metabolite features in the prepregnancy overweight and obesity group identified two filtered significant pathways: steroid hormone biosynthesis and neuroactive ligand‒receptor interaction pathways. In the steroid hormone biosynthesis pathway, the levels of several glucocorticoids (including corticosterone, 11-deoxycortisol, cortisol, testosterone, and 7α-hydroxytestosterone) were decreased in the prepregnancy overweight/obesity group. In addition to the physiological role of glucocorticoids in the healthy neuroendocrine development and maturation of fetuses and babies, glucocorticoids are essential to human health by regulating different physiological events in mature organs and tissues, such as glucose metabolism, lipid biosynthesis and distribution, food intake, thermogenesis, and mood and learning patterns [27]. Glucocorticoids have been considered as a link between adverse early-life conditions and the development of metabolic disorders in later life [28–30]. However, there is still much controversy regarding the role of maternal obesity in the fetal–steroid hormone biosynthesis pathway. Studies of maternal obesity animal models showed that corticosterone and cortisol levels were increased in the offspring of obese mothers [31, 32]. A study reported by Satu M Kumpulainen et al. showed that young adults born to mothers with higher early pregnancy BMIs show lower average levels of diurnal cortisol, especially in the morning [33]. Laura I. Stirrat et al. found that increased maternal BMI was associated with lower maternal cortisol, corticosterone, and 11-dehydrocorticosterone levels. However, there were no associations between maternal BMI and glucocorticoid levels in the cord blood [34]. The differences in the study protocols of these previous studies may explain the mixed findings, such as cortisol measured from peripheral blood, cord blood or saliva; variation in measurement time points; the number of samples. Although the effect of maternal obesity on fetal steroid hormone levels is controversial, dysregulation of glucocorticoids may be a plausible mechanism by which maternal obesity can increase the risk of metabolic disorders and mental health disorders in offspring.

The effect of excessive GWG on umbilical cord blood metabolites is different from that of maternal overweight and obesity. Compared with the control group, in the excessive GWG group, the level of thromboxane B2 was increased and the level of hippuric acid was decreased. Thromboxane B2, which is important in the platelet release reaction, is a stable, physiologically active compound formed in vivo from prostaglandin endoperoxides. Hippuric acid is an acyl glycine formed from the conjugation of benzoic acid with glycine. Several studies have confirmed that both thromboxane B2 and hippuric acid levels are associated with diet. Dietary fatty acids affect platelet thromboxane production [35–37]. In our study, several fatty acids (e.g., palmitic acid, stearic acid, behenic acid, and lignoceric acid) in the excessive GWG group were also increased, which may have led to the increase in thromboxane B2 levels. Hippuric acid can be detected after the consumption of whole grains and anthocyanin-rich bilberries [38, 39]. A healthy diet intervention increased the signals for hippuric acid to incorporate polyunsaturated fatty acids [38], and the low level of hippuric acid was associated with lower fruit-vegetable intakes [39]. Maternal overnutrition and unhealthy dietary patterns are the main reasons for excessive GWG [40, 41]. Therefore, we speculated that the differences in thromboxane B2 and hippuric acid between the excessive GWG and control groups were associated with maternal diet during pregnancy. The effect of these differential metabolites on the long-term metabolic health of offspring after birth needs further study.

Metabolomic pathway analysis of the cord blood metabolite features in the excessive GWG group identified that biosynthesis of unsaturated fatty acids was the filtered significant pathway. The levels of several fatty acids in this pathway were increased in the excessive GWG group, including long-chain saturated fatty acids (e.g., palmitic acid (C 16:0), stearic acid (C 18:0), behenic acid (C 22:0), and lignoceric acid (C 23:0)), monounsaturated fatty acids (erucic acid), and polyunsaturated fatty acids (e.g., DPA, DHA, arachidonic acid, and adrenic acid). Because perinatal fatty acid status can be influenced by maternal dietary modifications or supplementation [42], we speculated that maternal diet during pregnancy caused the difference in umbilical cord blood fatty acids between the excessive GWG and control groups. A large body of evidence from mechanistic studies supports the potential of fatty acids to influence later obesity. However, the possible mechanisms and observed relationships are complex and related to the types and patterns of fatty acids [43, 44]. Maternal dietary fatty acids have been found to induce hypothalamic inflammation, cause epigenetic changes, and alter the mechanisms of energy control in offspring [43]. Evidence from cell culture and rodent studies showed that polyunsaturated fatty acids might serve several complex roles in fetuses, including the stimulation and/or inhibition regulation of adipocyte differentiation [44]. The questions of whether lower n-6 long-chain polyunsaturated fatty acid levels or higher n-3 long-chain polyunsaturated fatty acid levels are of more relevance and whether the long-term effects differ with different offspring ages remain [44]. Although there is a biologically plausible case for the relevance of perinatal fatty acid status in later obesity risk, available data in humans suggest that the influence of achievable modification of perinatal n-3/n-6 status is not sufficient to influence offspring obesity risk in the general population [45]. Further studies seem justified to clarify the reasons.

The advantage of our present study is that we simultaneously analyzed the effects of prepregnancy overweight/obesity and excessive GWG on cord blood metabolites and explored their differences. In addition, to exclude the effect of hyperglycemia on cord blood metabolites, both women with prepregnancy diabetes mellitus and gestational diabetes mellitus were excluded from our study. The limitation of our study is that it was a single-center study with a small sample, especially in the prepregnancy overweight/obesity group. In the future, we can expand the sample size and conduct a subgroup analysis of the prepregnancy overweight/obesity group and analyze the differences in the effects of different degrees of obesity on cord blood metabolites. The prepregnancy overweight/obesity group can be further divided into an appropriate GWG group and an excessive GWG group, and the differences in the effects of these two groups on umbilical cord blood metabolites can be analyzed. Moreover, the dietary pattern of the pregnant woman could affect the production of cord blood metabolites. We did not investigate the dietary patterns of the mothers in this study, which is another limitation of this study. In future studies, we should investigate maternal dietary patterns as a very important confounding variable.

## Conclusions

In conclusion, our present study confirmed that both prepregnancy overweight/obesity and excessive GWG could affect umbilical cord blood metabolites, and they had different effects on these metabolites. Prepregnancy overweight and obesity affected the fetal steroid hormone biosynthesis pathway, while normal prepregnancy body weight but excessive GWG affected fetal fatty acid metabolism. This emphasizes the importance of preconception weight loss and maintaining an appropriate GWG, which are beneficial for the long-term metabolic health of offspring.

### Supplementary Information

**Supplementary Material 1.**

## Data Availability

Data sets generated during the current study are not publicly available but will be available from the corresponding author at a reasonable request. Responses to the request for the raw data will be judged by a committee including XXY and GHL.

## Abbreviations

GWG: Excessive gestational weight gain
UHPLC-MS/MS: Ultrahigh-performance liquid chromatography tandem mass spectrometry
T2DM: Type 2 diabetes mellitus
NAFLD: Nonalcoholic fatty liver disease
DOHaD: The developmental origins of health and disease
BMI: Body mass index
DM: Diabetes mellitus
GDM: Gestational diabetes mellitus
OGTT: Oral glucose tolerance test
LBW: Low birth weight
SD: Standard deviation
HMDB: The Human Metabolome Database
KEGG: Kyoto Encylopaedia of Genes and Genomes
PCA: Principal component analysis
PLS-DA: Partial least-squares discriminant analysis
VIP: Importance for the projection
FC: Fold change
IVF-ET: Invitro fertilization and embryo transfer
TG: Triglyceride
UA: Uric acid
DPA: Docosapentaenoic acid
DHA: Docosahexaenoic acid
GVAs: Gastric vagal afferents
