# Supplementary material for: The influence of maternal prepregnancy weight and gestational weight gain on the umbilical cord blood metabolome: a case–control study
Source: BMC Pregnancy Childbirth. 2024 Apr 22;24:297. doi: 10.1186/s12884-024-06507-x (PMC11034091; doi:10.1186/s12884-024-06507-x)
Supplement: Supplementary file 1 — Supplementary Material 1. [file 12884_2024_6507_MOESM1_ESM.docx]

**Supplementary Materials and Methods**

**Umbilical cord blood sample**

The umbilical cord blood samples (100 μL) were placed in the EP tubes and resuspended with prechilled 80% methanol by well vortex. Then the samples were incubated on ice for 5 min and centrifuged at 15,000 g, 4 °C for 20 min. Some of supernatant was diluted to final concentration containing 53% methanol by liquid chromatograph mass spectrometer (LC-MS) grade water. The samples were subsequently transferred to a fresh Eppendorf tube and then were centrifuged at 15,000 g, 4 °C for 20 min. Finally, the supernatant was injected into the LC-MS/MS system analysis.

**UPLC-MS/MS Analysis**

Ultra-high-performance liquid chromatography tandem mass spectrometry (UHPLC-MS/MS) analyses were performed using a Vanquish UHPLC system (Thermo Fisher, Germany) coupled with an Orbitrap Q Exactive ^TM^ HF mass spectrometer (Thermo Fisher, Germany) in Novogene Co., Ltd. (Beijing, China). Samples were injected onto a Hypesil Gold column (100×2.1 mm, 1.9 μm) using a 17-min linear gradient at a flow rate of 0.2 mL/min. The eluents for the positive polarity mode were eluent A (0.1% FA in water) and eluent B (Methanol). The eluents for the negative polarity mode were eluent A (5 mM ammonium acetate, pH 9.0) and eluent B (Methanol). The solvent gradient was set as follows: 2% B, 1.5 min; 2-85% B, 3 min; 85-100% B, 10 min; 100-2% B, 10.1 min; 2% B, 12 min. Q Exactive ^TM^ HF mass spectrometer was operated in positive/negative polarity mode with spray voltage of 3.5 kV, capillary temperature of 320 °C, sheath gas flow rate of 35 psi and aux gas flow rate of 10 L/min, S-lens RF level of 60, Aux gas heater temperature of 350 °C.

**Data Processing and Metabolite Identification**

The raw data files generated by UHPLC-MS/MS were processed using the Compound Discoverer 3.1 (CD3.1, Thermo Fisher) to perform peak alignment, peak picking, and quantitation for each metabolite. The main parameters were set as follows: retention time tolerance, 0.2 minutes; actual mass tolerance, 5ppm; signal intensity tolerance, 30 %; signal/noise ratio, 3; and minimum intensity, *et al*. After that, peak intensities were normalized to the total spectral intensity. The normalized data were used to predict the molecular formula based on additive ions, molecular ion peaks and fragment ions. And then peaks were matched with the mzCloud (https://www.mzcloud.org/), mzVault and MassList database to obtain the accurate qualitative and relative quantitative results. Statistical analyses were performed using the statistical software R (R version R-3.4.3), Python (Python 2.7.6 version), and CentOS (CentOS release 6.6). When data were not normally distributed, standardized according to the formula: sample raw quantitation value / (The sum of sample metabolite quantitation value / The sum of QC1 sample metabolite quantitation value) to obtain relative peak areas, and compounds whose CVs of relative peak areas in QC samples were greater than 30 % were removed, and finally, the metabolites' identification and relative quantification results were obtained.
